# Modelling Transcriptional Regulation with a Mixture of Factor Analyzers and Variational Bayesian Expectation Maximization

**DOI:** 10.1186/1687-4153-2009-601068

**Published:** 2009-04-12

**Authors:** Kuang Lin, Dirk Husmeier

**Affiliations:** 1 Biomathematics & Statistics Scotland (BioSS), Edinburgh EH93JZ, UK

## Abstract

Understanding the mechanisms of gene transcriptional regulation through analysis of high-throughput postgenomic data is one of the central problems of computational systems biology. Various approaches have been proposed, but most of them fail to address at least one of the following objectives: (1) allow for the fact that transcription factors are potentially subject to posttranscriptional regulation; (2) allow for the fact that transcription factors cooperate as a functional complex in regulating gene expression, and (3) provide a model and a learning algorithm with manageable computational complexity. The objective of the present study is to propose and test a method that addresses these three issues. The model we employ is a mixture of factor analyzers, in which the latent variables correspond to different transcription factors, grouped into complexes or modules. We pursue inference in a Bayesian framework, using the Variational Bayesian Expectation Maximization (VBEM) algorithm for approximate inference of the posterior distributions of the model parameters, and estimation of a lower bound on the marginal likelihood for model selection. We have evaluated the performance of the proposed method on three criteria: activity profile reconstruction, gene clustering, and network inference.

## 1. Introduction

Transcriptional gene regulation is a complex process that utilizes a network of interactions. This process is primarily controlled by diverse regulatory proteins called transcription factors (TFs), which bind to specific DNA sequences and thereby repress or initiate gene expression. Transcriptional regulatory networks control the expression levels of thousands of genes as part of diverse biological processes such as the cell cycle, embryogenesis, host-pathogen interactions, and circadian rhythms. Determining accurate models for TF-genes regulatory interactions is thus an important challenge of computational systems biology. Most recent studies of transcriptional regulation can be placed broadly in one of three categories.

Approaches in the first class attempt to build quantitative models to associate gene expression levels, as typically obtained from microarray experiments, with putative binding motifs on the gene promoter sequences. Bussemaker et al. [1] and Conlon et al. [2] propose a linear regression model for the dependence of the log gene expression ratio on the presence of regulatory sequence motifs. Beer and Tavazoie [3] cluster gene expression profiles in a preliminary data analysis based on correlation, and then apply a Bayesian network classifier to predict cluster membership from sequence motifs. Phuong et al. [4] use multivariate decision trees to find motif combinations that define homogeneous groups of genes with similar expression profiles. Segal et al. [5] cluster genes with a probabilistic generative model that systematically integrates gene expression profiles with regulatory sequence motifs.

A shortcoming of the methods in the first class is that the activities of the TFs are not included in the model. This limitation is addressed by models in the second class, which predict gene expression levels from both binding motifs on promoter sequences and the expression levels of putative regulators. Middendorf et al. [6, 7] approach this problem as a binary classification task to predict up- and down-regulation of a gene from a combination of a motif presence/absence indication and the discrete state of a putative regulator. The bidimensional regression trees of Ruan and Zhang [8] are based on a similar idea, but avoid the information loss inherent in the binary gene expression discretization. 

**Figure 1:**
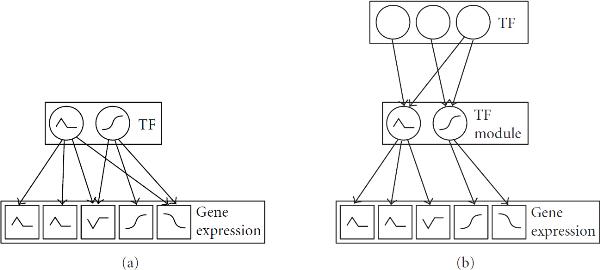
**Transcriptional regulatory network**. (a) A transcriptional regulatory network in the form of a bipartite graph, in which a small number of transcription factors (TFs), represented by circles, regulate a large number of genes (represented by squares) by binding to their promoter regions. The black lines in the square boxes indicate gene expression profiles, that is, gene expression values measured under different experimental conditions or for different time points. The black lines in the circles represent TF activity profiles, that is, the concentrations of the TF subpopulation capable of DNA binding. Note that these TF activity profiles are usually unobserved owing to posttranslational modifications, and should hence be included as hidden or latent variables in the statistical model. (b) A more accurate representation of transcriptional regulation that allows for the cooperation of several TFs forming functional complexes; this complex formation is particularly common in higher eukaryotes.

Transcriptional regulation is influenced by TF *activities*, that is the concentration of the TF subpopulation capable of DNA binding. The methods in the second class approximate the activities of TFs by their gene expression levels. However, TFs are frequently subject to post-translational modifications, which may affect their DNA binding capability. Consequently, gene expression levels of TFs contain only limited information about their actual activities. The methods in the third class address this shortcoming by treating TFs as latent or hidden components. The regulatory system is modelled as a bipartite network, as shown in Figure 1(a), in which high-dimensional output data are driven by low-dimensional regulatory signals. The high-dimensional output data correspond to the expression levels of a large number of regulated genes. The regulators correspond to a comparatively small number of TFs, whose activities are unknown. Various authors have applied latent variable models like principal component analysis (PCA), factor analysis (FA), and independent component analysis (ICA) to determine a low-dimensional representation of high-dimensional gene expression profiles; for example, Raychaudhuri et al. [9] and Liebermeister [10]. However, these approaches provide only a phenomenological modelling of the observed data, and the hidden components do not correspond to identified TFs. Liao et al. [11] and Kao et al. [12] address this shortcoming by including partial prior knowledge about TF-gene interactions, as obtained from Chromatin Immunoprecipitation (ChIP) experiments [13] or binding motif finding algorithms (e.g., Bailey and Elkan [14]; Hughes et al. [15]). Their network component analysis (NCA) is equivalent to a constrained maximum likelihood procedure in the presence of Gaussian noise and independent hidden components; the latter represent the TF activities. A major limitation of NCA is the fact that the constraints on the connectivity pattern of the bipartite network are rigid, which does not allow for the noise intrinsic to immunoprecipitation experiments or sequence motif detection. Sabatti and James [16] and Sanguinetti et al. [17] address this shortcoming by proposing an approach based on Bayesian factor analysis, in which prior knowledge about TF-gene interactions naturally enters the model in the form of a prior distribution on the elements of the loading matrix. Pournara and Wernisch [18] propose an alternative approach based on maximum likelihood, where the loading matrix is orthogonally rotated towards a target matrix of a priori known TF-gene interactions. All three approaches simultaneously reconstruct the structure of the bipartite regulatory network—represented by the loading matrix—and the TF activity profiles—represented by the hidden factors—from gene expression data and (noisy) prior knowledge about TF-gene interactions. In a recent generalization of these approaches, Shi et al. [19] have introduced a further latent variable to indicate whether a TF is transcriptionally or posttranscriptionally regulated.

Contrary to the methods in the first two classes, the methods in the third class do not incorporate interaction effects between TFs, though. This is a major limitation, since especially in higher eukaryotes transcription factors cooperate as a functional complex in regulating gene expression [20, 21]. Boulesteix and Strimmer [22] allow for this complex formation by proposing a latent variable model in which the latent components correspond to groups of TFs. However, their partial-least squares (PLS) approach does not provide a probabilistic model and hence, like NCA, does not allow for the noise inherent in TF binding profiles from immunoprecipitation experiments or sequence motif detection schemes.

In the present paper we aim to combine the advantages of the methods in the three classes summarized above. Like the approaches in the third class, our method is a latent variable model that allows for the fact that owing to post-translational modifications the true TF activities are unknown. Similar to the approaches of the first two classes, our model explicitly incorporates interactions among TFs. Inspired by Boulesteix and Strimmer [22], we aim to group individual TFs into TF modules, as illustrated in Figure 1(b). To allow for the noise inherent in both gene expression levels and TF binding profiles, we use a proper probabilistic generative model, like Sanguinetti et al. [17] and Sabatti and James [16]. Our work is based on the work of Beal [23]. We apply a mixture of factor analyzers model, in which each component of the mixture corresponds to a TF complex composed of several TFs. This approach allows for the fact that TFs are not independent. By explicitly including this in our model we would expect to end up with fewer parameters, and hence more stable inference. To further improve the robustness of this approach, we pursue inference in a Bayesian framework, which includes a model selection scheme for estimating the number of TF complexes. We systematically integrate gene expression data and TF binding profiles, and treat both as *data*. This appears methodologically more consistent than the approach in Sanguinetti et al. [17] and Sabatti and James [16], where TF binding data are treated as *prior knowledge*. Our paper is organized as follows. In Section 2 we review Bayesian factor analysis applied to modelling transcriptional regulation. In Section 3 we discuss how TF complexes and interaction effects among TFs can be modelled with a mixture of factor analyzers. The data used for the evaluation of the method are described in Section 4. Section 5 provides three types of results related to the reconstruction of the unknown TF activity profiles are discussed in Section 5.1, gene clustering is discussed in Section 5.2, and the reconstruction of the transcriptional regulatory network is discussed in Section 5.3. We conclude our paper in Section 6 with a summary and a brief outlook on future work.

## 2. Background

In this section, we will briefly review the application of Bayesian factor analysis to transcriptional regulation. To keep the notation simple, we use the same letter  for every probability distribution, even though they might be of different functional forms. The form of  will become clear from its argument, with  and  denoting different distributions (strictly speaking, this should be written as  and ). Variational distributions will be written as . We do not distinguish between random variables and their realization in our notation. However, we do distinguish between scalars and vectors/matrices, using bold-face letters for the latter, and using the superscript "" to denote transposition.

Given the expression levels of  genes at the th experimental condition, the objective of factor analysis (FA) is to model correlations in high-dimensional data  by correlations in a lower-dimensional subspace of unobserved or latent vectors , which are assumed to have a zero-mean, unit-variance Gaussian distribution. The model assumes that the latent vectors  are linearly mapped into the high-dimensional space via a so-called loading matrix , then translated by , and finally subjected to additive noise from a zero-mean Gaussian distribution with diagonal covariance matrix . Mathematically, this procedure can be summarized as follows: (1)(2)

where  denotes a multivariate Gaussian distribution with mean vector  and covariance matrix  is a zero-vector, and  denotes the identity matrix. This probabilistic generative model was first proposed by Ghahramani and Hinton [24]. Note that in the context of gene regulation, the vector  corresponds to the gene expression profile in experimental condition , the latent vector  denotes the (unknown) TF activities in the same experimental condition, and the elements of the loading matrix  represent the strengths of the interactions between the TFs and the regulated genes. Integrating out the latent vectors , it can be shown (see, for instance, Nielsen [25]) that (3)

where, from (A.3) and (A.25) (4)

The likelihood of the data , where  is the number of experimental conditions or time points, is given by (5)

One can then, in principle, estimate the parameters  in a maximum likelihood sense, using for instance the EM algorithm proposed in Ghahramani and Hinton [24] and Nielsen [25]. However, the maximum likelihood configuration is not uniquely determined owing to two intrinsic identifiability problems. First, there is a scale identifiability problem: multiplying the loading matrix  by some factor  and dividing the latent variables  by the same factor will leave (A.3) invariant. Second, subjecting the latent variables  to an orthogonal transformation  will leave the covariance matrix in (3) invariant, since . Pournara and Wernisch [18] deal with this invariance by applying a varimax transformation to rotate the loading matrix  towards maximum sparsity. The justification of this approach, which we investigated in our empirical evaluation to be discussed in Section 5, is that gene regulatory networks are usually sparsely connected, rendering sparse loading matrices  biologically more plausible. An alternative approach to deal with this invariance, which also allows the systematic integration of biological prior knowledge, is to adopt a Bayesian approach. Here, the parameters  are interpreted as random variables, for which prior distributions are defined. While the likelihood shows a ridge owing to the invariance discussed above, the posterior distribution does not (unless the prior is uninformative), which solves the identifiability problem. The most straightforward approach, chosen for instance in Nielsen [25], Ghahramani and Beal [26] and Beal [23], is a set of spherical Gaussian distributions as a prior distribution for the column vectors in , where  is the number of latent factors: (6)

and a conjugate prior on the hyperparameters  in the form of a gamma distribution; see (20). This approach shrinks the elements of the loading matrix  to zero and is therefore similar in spirit to the varimax rotation mentioned above. A more sophisticated approach, which allows a more explicit inclusion of biological prior knowledge about TF-gene interactions, was proposed in Sanguinetti et al. [17] and Sabatti and James [16], based on the work of West [27]. The models differ in various details, but the generic idea can be described as follows. The loading matrix element , which indicates the strength of the regulatory interaction between TF  and gene , has the prior probability (7)

where  is the unit point mass at zero (the delta distribution), and  denotes the prior probability of  to be different from zero. The precision hyperparameter  is given a gamma distribution with hyperparameters  and , Gamma(; see (20). For the practical implementation, a set of binary auxiliary variables  is introduced, which indicate the presence or absence of an interaction: (8)

The prior probability on the matrix of auxiliary variables  is given by (9)

where the values of  allow the inclusion of prior knowledge about TF-gene regulatory interactions, as obtained, for example, from immunoprecipitation experiments or sequence motif finding algorithms.

The objective of Bayesian inference is to learn the posterior distribution of the model parameters and latent variables. Since this distribution does not have a closed form, approximate procedures have to be adopted. Sabatti and James [16] follow a Markov chain Monte Carlo (MCMC) approach based on the collapsed Gibbs sampler. Here, each of the parameters  and  and latent variables  and  is sampled separately from a closed-form distribution that depends on sufficient statistics defined by the other parameters/latent variables, and the procedure is iterated until some convergence criterion is met. Sanguinetti et al. [17] follow an alternative approach based on Variational Bayesian Expectation maximization (VBEM), where the joint posterior distribution of the parameters and latent variables is approximated by a product of model distributions for which closed-form solutions can be obtained; see Section A.1 of the appendix.

## 3. Method

The Bayesian FA models discussed in the previous section aim to explain changes in gene expression levels from the activities of TFs, modelled as the hidden factors or latent variables . This does not allow for the fact that in eukaryotes TFs usually work in cooperation and form complexes [20], and that gene regulation should be addressed in terms of cis-regulatory modules rather than individual TF-gene interactions. In the present paper, we address this shortcoming by applying a mixture of factor analyzers (MFAs) approach. Probabilistic mixture models are discussed in [42, Chapter 9], and the application to factor analysis models is discussed, for instance, in McLachlan et al. [28]. We used a slight variation of the mixture of factor analyzers (MFAs) approach proposed in Ghahramani and Beal [26] and Beal [23]. Each component of the mixture represents a TF complex. TF complexes are assumed to bind to the gene promoters competitively, that is, each gene is regulated by a single TF complex. Hence, while a gene can be regulated by several TFs, these TFs do not act individually, but exert a combined effect on the regulated gene via the TF complex they form. In terms of modelling, our approach results in a dimension and complexity reduction similar to the partial least squares method proposed in Boulesteix and Strimmer [22], with the difference that the approach proposed in the present paper has the well-known advantages of a probabilistic generative model, like improved robustness to noise and the provision of an objective score for model selection and inference. Consider the mixture model (10)

where  is a discrete random variable that indicates the component from which  has been generated, and each component probability density  is given by (3).  is a prior probability distribution on the components, defined by the vector of component proportions  via . The component proportions are given a conjugate prior in the form of a symmetric Dirichlet distribution with hyperparameter , where (11)

As discussed in Section 2, (10) offers a way to relax the linearity constraint of FA by means of tiling the data manifold. One approach would be for  to represent the vector of gene expression values under experimental condition , and each experimental condition to be assigned to one of  classes. However, this method would not achieve the grouping of genes according to transcriptional modules. We therefore transpose the data matrix , where  is the number of experimental conditions or time points, to obtain the new representation , where  is the number of genes, and  denotes the -dimensional column vector with expression values for gene  under all experimental conditions. As we will be using this representation consistently in the remainder of the paper, we will not make the transposition () explicit in the notation. Note that in this new representation, (10) provides a natural way to assign genes to transcriptional modules, represented by the various components of the mixture. Recall that in (A.3), the dimension of the hidden factor vector  reflects the number of TFs regulating the genes. In the proposed MFA model, the hidden factors are related to TF complexes. Since each gene is assumed to be regulated by a single complex, as discussed above, the hidden factor vector becomes a scalar: . The loading matrix  in (A.3) becomes a vector of the same dimension as  and represents the TF complex activity profile (covering the experimental conditions or time points for which gene expression values have been collected in ). We write this as . Equations (A.3) and (A.25) thus become: (12)(13)

in which  defines a diagonal covariance matrix, as before. This can be rewritten as: (14)

For (3) we now get: (15)

which completes the definition of (10). Recall that in (A.3), the loading matrix  provides a mechanism for including biological prior knowledge about TF-gene interactions; this approach, which was pursued in Sabatti and James [16], is affected by the mixture prior of (7)–(9). However, like gene expression levels, indications about TF-gene interactions are usually obtained from microarray-type experiments (ChIP-on-chip immunoprecipitation experiments). It appears methodologically somewhat inconsistent to treat these two types of data differently, and to treat gene expression levels as proper data, while treating TF binding data as prior knowledge. In our approach, we therefore seek to treat both types of data on an equal footing. Denote by  the expression profile of gene , that is, the vector containing the expression values of gene  for the selected experimental conditions or time points. In other words:  is the expression level of gene  in experimental condition  (or at time point ). Denote by  the TF binding profile of gene . This is a vector indicating the binding affinities of a set of TFs for gene . Expressed differently,  is the measured strength with which TF  binds to the promoter of gene . In our approach, we concatenate these vectors to obtain an expanded column vector : (16)

In practice, gene expression and TF binding profiles will usually be differently distributed. The former tend to be approximately log-normally distributed, while for the latter we tend to get -values distributed in the interval . It will therefore be advisable to standardize both types of data to Normal distributions. For gene expression values this implies a transformation to log ratios (or, more accurately, the application of the mapping discussed in Huber et al. [29]). -values are transformed via , where  is the cumulative distribution function of the standard Normal distribution. If  is properly calculated as a genuine -value, then under the null hypothesis of no significant TF binding,  will be normally distributed. The concatenation expressed in (16) implies a corresponding concatenation of the parameter vectors  and : (17)

and the hyperparameters: (18)

where  and  define the prior distributions on the parameters, as discussed below. The resulting model can be interpreted as follows:  represents the composition of the th transcriptional module, that is, it indicates which TFs bind cooperatively to the promoters of the regulated genes.  allows for perturbations that result, for example, from the temporary inaccessibility of certain binding sites or a variability of the binding affinities caused by external influences.  is the "background" gene expression profile.  represents the activity profile of the th transcriptional module, which modulates the expression levels of the regulated genes.  describes the gene-specific susceptibility to transcriptional regulation, that is, to what extent the expression of the th gene is influenced by the binding of a transcriptional module to its promoter. Naturally, this information is contained in the expression profiles  and TF binding profiles  of the genes that are (softly) assigned to the th mixture component, while (12) and (13) provide a mechanism to allow for the noise in the data.

Here is an alternative interpretation of our model, which is based on the assumption that a variation of gene expression is brought about by different TFs binding in different proportions to the promoter. In the ideal case, genes with the same TFs binding in identical proportions to the promoter should have identical gene expression profiles; this is expressed in our model by  (the proportions of TFs binding to the promoter), and  (the "background" gene expression profile associated with the idealized binding profile of the TFs). Obviously, this model is oversimplified. There are two reasons why gene expression profiles might deviate from this idealized profile. The first reason is measurement errors and stochastic fluctuations unrelated to the TFs. These influences are incorporated in the additive term  in (12). The second reason is variations in the TF binding affinities, their activities and binding capabilities. These variations are captured by the vector . The changes in the way TFs bind to the promoter will result in deviations of the gene expression profiles from the idealized "background" distribution; these deviations are defined by the vector . We assume that if the deviation of the TF binding profiles from the idealized binding profile  is small, the deviation from the "background" gene expression profile  will be small. Conversely, if the TFs show a considerable deviation from the idealized binding profile , then the gene expression profile will show a substantial deviation from the idealized expression profile . We therefore scale both  and  by the same gene-specific factor ; this enforces a hard association between the two effects described above. Weakening this association would be biologically more realistic, but at the expense of increased model complexity.

To complete the specification of the model, we need to define prior distributions for the various parameter groups. In the present paper we follow Beal [23] and impose prior distributions on all parameters that scale with the complexity of the model, that is, the number of mixture components . These are the factor loadings  and displacement vectors . The idea is that the proper Bayesian treatment, that is, the integration over these parameters, is essential to prevent over-fitting. Since the number of degrees of freedom in  does not depend on the complexity of the model, integrating over these parameters is less critical. In the present approach we therefore follow the simplification suggested in Beal [23] and treat  as a parameter group to be estimated by maximization of  in (22), see (A.24), rather than a random variable with its own prior distribution. Like in (6), a hierarchical prior is used for the factor loadings : (19)

with gamma distributions for the precision hyperparameters : (20)

**Figure 2:**
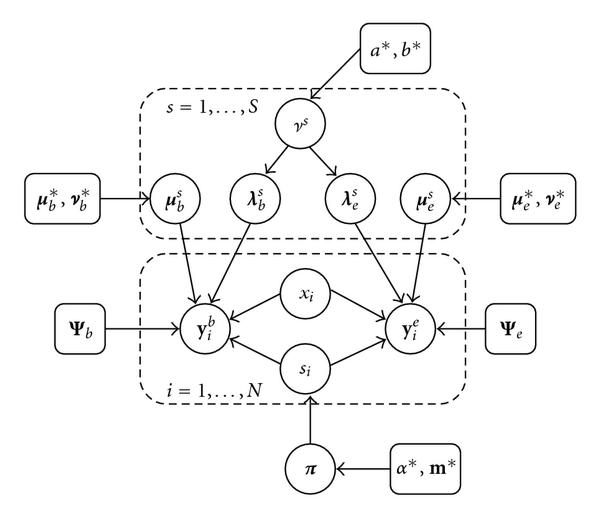
**Bayesian mixture of factor analyzers (MFA) model applied to transcriptional regulation**. The figure shows a probabilistic independence graph of the Bayesian mixture of factor analyzers (MFA) model proposed in Section 3. Variables are represented by circles, and hyperparameters are shown as square boxes in the graph.  components (factor analyzers), each with their own parameters  and , are used to model the expression profiles  and TF binding profiles  of  genes. The factor loadings  have a zero-mean Gaussian prior distribution, whose precision hyperparameters  are given a gamma distribution determined by  and . The analyzer displacements  and  have Gaussian priors determined by the hyperparameters  and , respectively. The indicator variables  select one out of  factor analyzers, and the associated latent variables or factors  have normal prior distributions. The indicator variables  are given a multinomial distribution, whose parameter vector , the so-called mixture proportions, have a conjugate Dirichlet prior with hyperparameters .  and  are the diagonal covariance matrices of the Gaussian noise in the expression and binding profiles, respectively. A dashed rectangle denotes a plate, that is an iid repetition over the genes  or the mixture components , respectively. The biological interpretation of the model is as follows.  represents the composition of the th transcriptional module, that is, it indicates which TFs bind cooperatively to the promoters of the regulated genes.  allows for perturbations that result, for example, from the temporary inaccessibility of certain binding sites or a variability of the binding affinities caused by external influences.  is the background gene expression profile.  represents the activity profile of the th transcriptional module, which modulates the expression levels of the regulated genes.  describes the gene-specific susceptibility to transcriptional regulation, that is, to what extent the expression of the th gene is influenced by the binding of a transcriptional module to its promoter. A complete description of the model can be found in Section 3.

A Gaussian prior with mean  and precision matrix  is placed on the factor analyzer displacements : (21)

where  is a square matrix that has the vector  in its diagonal, and zeros everywhere else. The corresponding probabilistic graphical model is shown in Figure 2.

The objective of Bayesian inference is to estimate the posterior distribution of the parameters and the marginal posterior probability of the model (i.e., the number of components in the mixture). The two principled approaches to this end are MCMC and VBEM. A sampling-based approach based on MCMC has been proposed in Fokoué and Titterington [30]. A VBEM approach has been proposed in Ghahramani and Beal [26] and Beal [23]. In the present work, we follow the latter approach. As briefly reviewed in the appendix, Section A.1, the VBEM approach is based on the choice of a model distribution that factorizes into separate distributions of the parameters and latent variables: , where  and . Following Beal [23], we assume the further factorization of the distribution of the parameters : , where  and . In generalization of (A.1) and (A.2) we can now derive the following lower bound on the marginal likelihood : (22)

where , , and all other symbols are defined in Figure 2 and in the text; see [23], equation (4.29)]. The variational E- and M-steps of the VBEM algorithm are derived as in Section A.1 by setting to zero the functional derivatives of  with respect to the different (hyper-)parameters and latent variables under consideration of possible normalization constraints, along the line of (A.4)–(A.7). The derivations can be found in Beal [23]. A summary of the update equations is provided in the appendix, Section A.2. The various (hyper-)parameters and latent variables are updated according to these equations iteratively, assuming the variational distributions  for the other (hyper-)parameters and latent variables are fixed. The algorithm is iterated until a stationary point of  is reached.

The final issue to address is model selection, that is, selecting the number of mixture components . Following Beal [23], we have not placed a prior distribution on , but instead have placed a symmetric Dirichlet prior over the mixture proportions ; see (11). Equation (22) provides a lower bound on the marginal likelihood , where the model  is defined by the number of mixture components . In order to navigate in the space of different model complexities, we use the scheme of birth and death moves proposed in Beal [23]. This scheme can be seen as the VBEM equivalent to reversible jump MCMC [31]. Via a birth or a death move, a component is removed from or introduced into the mixture model, respectively. The VBEM algorithm, outlined in the present section and stated in more detail in the appendix, Section A.2, is then applied until a measure of convergence is reached. On convergence, the move is accepted if  of (22) has increased, and rejected otherwise. Another birth/death proposal is then made, and the procedure is repeated until no further proposals are accepted. Further details of this birth/death scheme can be found in Beal [23]. Note that these birth and death moves also help avoid local maxima in , in a similar manner as discussed in Ueda et al. [32].

## 4. Data

We tested the performance of the proposed method on both simulated and real gene expression and TF binding data. The first approach has the advantage that the regulatory network structure and the activities of the TF complexes are known, which allows us to assess the prediction performance of the model against a known gold standard. However, the data generation mechanism is an idealized simplification of real biological processes. We therefore also tested the model on gene expression data and TF binding profiles from *Saccharomyces cerevisiae*. Although *S. cerevisiae* has been widely used as a model organism in computational biology, we still lack any reliable gold standard for the underlying regulatory network, and therefore need to use alternative evaluation criteria, based on out-of-sample performance. We will describe the data sets in the present section, and discuss the evaluation criteria together with the results in Section 5.

### 4.1. Synthetic Gene Expression and TF Binding Data

We generated synthetic data to simulate both the processes of transcriptional regulation as well as noisy data acquisition. We started from the activities of the TF protein complexes that regulate the genes by binding to their promoters. Note that owing to post-translational modifications these activities are usually not amenable to microarray experiments and therefore remain hidden. The advantage of the synthetic data is that we can assess to what extent these activities can be reconstructed from the gene expression profiles of the regulated genes.

**Figure 3:**
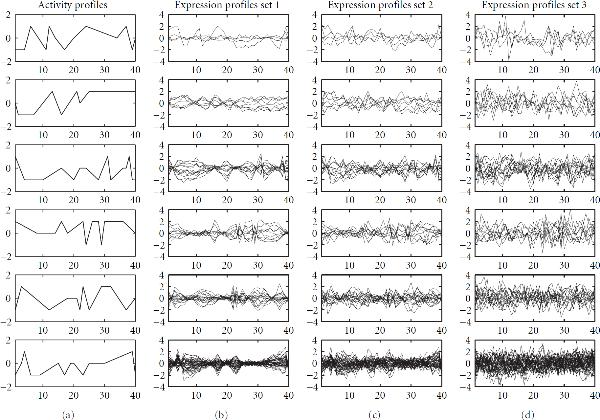
**Simulated TF activity and expression profiles**. (a) Simulated activity profiles of six hypothetical TF modules. The other panels show simulated expression profiles of the genes regulated by the corresponding TF module (in the same row). From left to right, the three sets have the corresponding observational noise levels of  and . The vertical axes show the activity levels (a) or relative log gene expression ratios (other panels), respectively, which are plotted against 40 hypothetical experiments or time points, represented by the horizontal axes.

Figure 3(a) shows the activity profiles , of 6 TF modules for 40 hypothetical experimental conditions or time points. Gene expression profiles (by *gene expression profile* we mean the vector of log gene expression ratios with respect to a control)  were given by (23)

where  represents stochastic fluctuations and dynamic noise intrinsic to the biological system, and  represents observational noise introduced by measurement errors. Here,  is the unit matrix. The expression profiles of 90 genes generated from (23) are shown in the right panels of Figure 3. The algorithms were tested with expression profile sets of three different noise levels: ,  or . They were also tested with expression profile sets of different lengths (numbers of time points or experimental conditions). The first 10, 20 or 40 time points were used.

Here we have assumed that each gene is regulated by a single TF complex. Note, however, that an individual TF can be involved in more than one TF module and therefore contribute to the regulation of different subsets of genes, as illustrated in Figure 1. Recall that TF modules are protein complexes composed of various TFs. In practice, we usually have only noisy indications about protein complex formations (e.g., from yeast 2-hybrid assays), and binding data are usually available for individual TFs (from binding motif similarity scores or immunoprecipitation experiments). In our simulation experiment we therefore assumed that the composition of the TF complexes was unknown, and that noisy binding data were available for individual TFs, as described shortly. 

**Figure 4:**
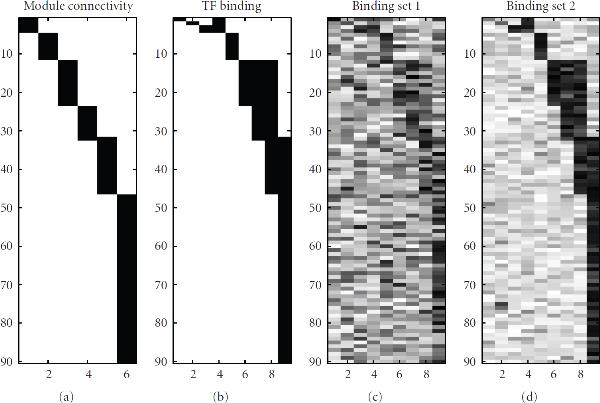
**Simulated TF binding data**. The figure shows simulated TF binding data. The vertical axis in each subfigure represents the 90 genes involved in the regulatory network. From left to right: (a) The binary matrix of connectivity between the 6 TF modules (horizontal axis) and the 90 genes, where black entries represent connections. Each module is composed of one or several TFs. (b) The real binding matrix between TFs (horizontal axis) and genes (vertical axis), with black entries indicating binding. (c), (d) The noisy binding data sets used in the synthetic study, with darker entries indicating higher values. Details can be found in Section 4.1.

**Figure 5:**
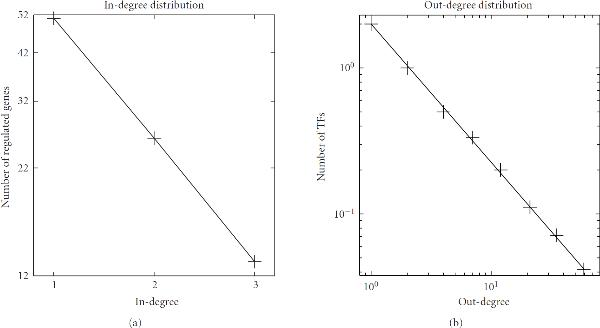
**In- and out-degree distributions of the simulated TF binding data**. (a) The arriving connectivity distribution (in-degree distribution). The number of genes regulated by  TFs follows an exponential distribution of  for in-degree . (b) The departing connectivity distribution (out-degree distribution). The number of TFs per  follows the power-law distribution of  for out-degree . Note that an exponential distribution is indicated by a linear relationship between  and  in a log-linear representation (a), whereas a distribution consistent with the power law is indicated by a linear dependence between  and  in a double logarithmic representation (b).

To group the TFs into modules when designing the synthetic TF binding set, we followed Guelzim et al. [33] and modelled the in-degree with an exponential distribution, and the out-degree with a power-law distribution. In particular, we chose the power-law distribution of  for the out-degree. The in-degree followed the exponential distribution of . The results are shown in Figure 5. In the binding matrix, 9 TFs are connected to 90 genes via 142 edges, as shown in Figure 4(b).

In the real world, TF binding data—whether obtained from gene upstream sequences via a motif search or from immunoprecipitation experiments—are not free of errors, and we therefore modelled two noise scenarios for two different data formats. In the first TF binding set, the non-binding elements were sampled from the beta distribution  and the binding elements from . For the second TF binding set, we chose  and  correspondingly. The resulting TF binding patterns are shown in Figures 4(c), 4(d).

### 4.2. Gene Expression and TF Binding Data from Yeast

For evaluating the inference of transcriptional regulation in real organisms, we chose gene expression and TF binding data from the widely used model organism *Saccharomyces cerevisiae* (baker's yeast). For the clustering experiments, we combined ChIP-chip binding data of 113 TFs from Lee et al. [34] with two different microarray gene expression data sets. From the Spellman set [35], the expression levels of 3638 genes at 24 time points were used. From the Gasch set [36], the expression values of 1993 genes at 173 time points were taken. For evaluating the regulatory network reconstruction, we used the gene expression data from Mnaimneh et al. [37] and the TF binding profiles from YeastTract [38]. YeastTract provides a comprehensive database of transcriptional regulatory associations in *S. cerevisiae*, and is publicly available from http://www.yeastract.com/. Our combined data set thus included the expression levels of 5464 genes under 214 experimental conditions and binary TF binding patterns associating these genes with 169 TFs. 

## 5. Results and Discussion

We have evaluated the performance of the proposed method on three criteria: activity profile reconstruction, gene clustering, and network inference. The objective of the first criterion, discussed in Section 5.1, is to assess whether the activity profiles of the transcriptional regulatory modules can be reconstructed from the gene expression data. The second criterion, discussed in Section 5.2, tests whether the method can discover biologically meaningful groupings of genes. The third criterion, discussed in Section 5.3, addresses the question of whether the proposed scheme can make a useful contribution to computational systems biology, where one is interested in the reconstruction of regulatory networks from diverse sources of postgenomic data. We have compared the proposed MFA-VBEM approach with various alternative methods: the partial least squares approach proposed of Boulesteix and Strimmer [22], maximum likelihood factor analysis, effected with the EM algorithm of Ghahramani and Hinton [24], and Bayesian factor analysis, using the Gibbs sampling approach of Sabatti and James [16]. We did not include network component analysis (NCA), introduced by Liao et al. [11], in our comparison. NCA effectively solves a constrained optimization problem, which only has a solution if the following three criteria are satisfied: (i) the connectivity matrix  must have full-column rank; (ii) each column of  must have at least  zeros, where  is the number of latent nodes; (iii) the signal matrix  must have full rank. These restrictions also apply to the more recent algorithmic improvement proposed in Chang et al. [40]. These regularity conditions were not met by our data. In particular, the absence of zeros in our connectivity matrices violated condition (ii), causing the NCA algorithm to abort with an error. An overview of the methods included in our comparative evaluation study is provided in Table 1.

**Table 1:** Overview of methods.

PLS	The partial least squares approach proposed by Boulesteix and Strimmer [22], using the software provided by the authors. Note that the method treats TF-gene interactions as fixed constants that cannot be changed in light of the gene expression data. Hence, this approach cannot be used for network reconstruction and was only applied for reconstructing the TF activity profiles.
FA	Maximum likelihood factor analysis, effected with the EM algorithm of Ghahramani and Hinton [24] and a subsequent varimax rotation [39] of the loading matrix towards maximum sparsity, as proposed in Pournara and Wernisch [18].

BFA-Gibbs	Bayesian factor analysis of Sabatti and James [16], trained with Gibbs sampling. The TF regulatory network is obtained from the posterior expected loading matrix via (A.32) and (A.35).

MFA-VBEM	The proposed mixture of factor analyzers model, shown in Figure 2 and discussed in Section 3, trained with variational Bayesian Expectation Maximization. The approach is based on the work of Beal [23], with the extension described in the text. The TF regulatory network is obtained from (24) and (25) for the curation and prediction tasks, respectively.

An overview of the methods compared in our study with a brief description of how the TF regulatory network was obtained.

### 5.1. Activity Profile Reconstruction

Since TF activity profiles are not available for real data, we used the synthetic data of Section 4.1 to evaluate the profile reconstruction performance of the model. We have compared the proposed MFA-VBEM model with the partial least-squares (PLS) approach of Boulesteix and Strimmer [22], and with the Bayesian factor analysis model using Gibbs sampling (BFA-Gibbs), as proposed in Sabatti and James [16].

The PLS approach of Boulesteix and Strimmer [22] is formally equivalent to the FA model of equation (A.3). However, the -by- loading matrix , which linearly maps  latent variables onto  genes, is decomposed into two matrices: an -by- matrix describing the interactions between  TFs and  genes, and an -by- matrix defining how the TFs interact to form modules; see Figure 1(b). The elements of the first matrix are fixed, taken from TF binding data (e.g., immunoprecipitation experiments or binding motifs). In the present example, the binding matrices of Figures 4(c), 4(d) were used. The elements of the second matrix are optimized so as to minimize the sum-of-squares deviation between the measured and reconstructed gene expression profiles subject to an orthogonality constraint for the latent profiles. These latent profiles are the predicted activity profiles of the TF modules. A cross-validation approach can in principle be used to optimize the number of TF modules . However, for ease of comparability of the reconstructed activity profiles with those obtained with the other methods we set  to the correct number of TF modules: . We carried out the evaluation using the software provided in Boulesteix and Strimmer [22], using the default parameters.

The BFA-Gibbs method of Sabatti and James [16] corresponds to a Bayesian FA model with a mixture prior on the elements of the loading matrix , which incorporates the information from immunoprecipitation experiments or binding motif search algorithms. In other words, the TF binding data, which in the present evaluation were the binding matrices of Figure 4, enter the model via the prior on , using (7)–(9). We sampled all parameters with the Gibbs sampling method of Sabatti and James [16], using the authors' programs, and applying standard diagnostic tools [41] to test for convergence of the Markov chains. The predicted activity profiles are the posterior averages of the latent factor profiles, computed from (4) in Sabatti and James [16].

For the proposed MFA-VBEM model, the activity profile of the th TF module is given by , the posterior average of , where  is the loading vector associated with the th module, and the posterior average  is obtained with the VBEM algorithm, using (A.17). The birth and death moves of the VBEM scheme, explained in Section 3, allow an estimation of the marginal posterior probability of the number of TFs, , which was found to peak at the correct value of . For a comparison with the alternative approaches, the simulations were repeated with the number of modules fixed at this value.

Table 2 shows a comparison of the reconstruction accuracy in terms of the mean absolute Pearson correlation between the true and estimated TF module activity profiles. It is seen that BFA-Gibbs and the proposed MFA-VBEM scheme consistently outperform PLS. The comparatively poor performance of PLS, which has been independently reported in Pournara and Wernisch [18], is a consequence of the fact that PLS lacks any mechanism to deal with the noise inherent in the TF binding profiles. In other words, the noisy TF binding data of Figure 4 are taken as true fixed TF-gene interactions, and there is no mechanism to adjust them in light of the gene expression data. This shortcoming is addressed by BFA-Gibbs and MFA-VBEM, which both allow for the noise inherent in the TF binding data.

**Table 2:** Reconstruction of TF complex activity profiles.

Method	B1	N1	N2	N3
PLS	L1	0.52	0.53	0.52
BFA		0.87	0.69	0.76
MFA		0.77	0.80	0.73

PLS	L2	0.52	0.52	0.52
BFA		0.84	0.68	0.59
MFA		0.89	0.71	0.60

PLS	L3	0.53	0.52	0.52
BFA		0.90	0.75	0.56
MFA		0.94	0.87	0.40

Method	B2	N1	N2	N3

PLS	L1	0.53	0.52	0.52
BFA		0.92	0.89	0.78
MFA		0.88	0.83	0.71

PLS	L2	0.52	0.51	0.52
BFA		0.83	0.72	0.72
MFA		0.95	0.85	0.71

PLS	L3	0.52	0.51	0.52
BFA		0.90	0.73	0.67
MFA		0.98	0.94	0.63

The mean absolute correlation coefficient between the true and inferred activity profiles, averaged over the 6 synthetic activity profiles of Figure 3. N1, N2 and N3 refer to the three noise levels of  and . L1, L2, and L3 refer to the expression profile lengths being 10, 20 and 40. B1 and B2 refer to the two different binding data sets with different levels of noise. Details are described in Section 4.1. Three methods have been compared: the partial least squares (PLSs) approach of Boulesteix and Strimmer [22]; the Bayesian factor analysis (BFA) model with Gibbs sampling, as proposed in Sabatti and James [16]; and the MFA model trained with VBEM, as described in Section 3.

A comparison between BFA-Gibbs and MFA-VBEM shows that BFA-Gibbs tends to outperform MFA-VBEM when the expression profiles are short (length L1) or when the noise level is high (N3). This could be a consequence of the different inference schemes ("VBEM" versus "Gibbs"). Short expression profiles and high noise levels lead to diffuse posterior distributions of the parameters. Variational learning—as opposed to Gibbs sampling—is known to lead to a systematic underestimation of the posterior variation [42], which could be a disadvantage here. However, MFA-VBEM consistently outperforms BFA-Gibbs on the longer expression profiles with lengths L2 and L3, and the lower noise levels N1 and N2. We would argue that this improvement in the performance is a consequence of the more parsimonious model ("MFA") that results when allowing for the fact that TFs are non-independent, which leads to greater robustness of inference and reduced susceptibility to over-fitting.

### 5.2. Gene Clustering

Following up on the seminal work of Eisen et al. [45], there has been considerable interest in clustering genes based on their expression patterns. The premise is based on the guilt-by-association hypothesis, according to which similarity in the expression profiles might be indicative of related biological functions. Although the main purpose of the proposed MFA-VBEM method is not one of clustering, it is straightforward to apply it to this end by using the model mixture proportions , which are obtained from the VBEM scheme via (A.22), as indicators of class membership. A convenient feature of the MFA-VBEM scheme is the fact that the number of clusters is identical to the number of mixture components in the model. This number is automatically inferred from the data using the model selection scheme based on birth-death moves, as described in Section 3. MFA-VBEM also allows for a straightforward integration of gene expression profiles with TF binding data.

We applied the MFA-VBEM method to the gene expression and TF binding data of *S. cerevisiae*, described in Section 4.2. For comparison, we also applied two standard clustering algorithms: K-means and hierarchical agglomerative average linkage clustering (see, e.g., Hastie et al. [46]). We used the implementation of these two algorithms in the Bioinformatics Toolbox of MATLAB (version 7.3.0), using default parameters and the default distance measure of 1 minus the absolute Pearson correlation coefficient. Five randomly chosen initial starting points were chosen for each application of K-means, and the most compact cluster formation found was recorded. For hierarchical clustering, we cut the dendrogram at such a distant from the root that the number of resulting clusters equalled the number of clusters used for MFA-VBEM and K-means. Note that unlike the proposed MFA-VBEM approach, K-means and average linkage clustering do not infer the number of clusters automatically from the data. To ensure comparability of the results we therefore set the number of clusters to be identical to the number of mixture components inferred with the MFA-VBEM method. We further included COSA [43] as a more advanced clustering algorithm in our comparison. The idea of clustering objects on subsets of attributes (COSA) is to detect subgroups of objects that preferentially cluster on subsets of the attribute variables rather than on all of them simultaneously. The relevant attribute subsets for each individual cluster can be different or partially overlap with other clusters. The attribute subsets are automatically selected by the algorithm via a weighting scheme that attempts to trade off two effects: (1) the objective to identify homogeneous and coherent clusters, and (2) the influence of an entropic regularization term that penalizes small subset sizes. In our study, we used the R program written by the authors, which is available from http://www-stat.stanford.edu/~jhf/COSA.html, using the default settings of the parameters. Clusters were obtained from the dendrogram in the same way as for hierarchical agglomerative average linkage clustering, subject to the constraint of having at least three genes in a cluster. Finally, we included Plaid model clustering [44] in our comparative evaluation study. Plaid model clustering is a non-mutually exclusive clustering approach, which allows a gene to have different cluster memberships. For the practical computation we used the Plaid (TM) software copyrighted by Stanford University, which is freely available from the following website: http://www-stat.stanford.edu/~owen/plaid/.

In order to evaluate the predicted clusters with respect to their biological plausibility, we tested them for significant enrichment of gene ontology (GO) annotations. To this end, we used the GO terms from the Saccharomyces genome database (SGD), which are publicly available from http://www.yeastgenome.org/. We assessed the enrichment for annotated GO terms in a given gene cluster with the program Ontologizer [47], using the default parameters. Given a population of genes with associated GO terms, Ontologizer associates each GO term with a -value. To correct for multiple testing, we controlled the family-wise type-I error conservatively with the Bonferroni correction, using a standard threshold at the 5% significance level. We called a gene cluster "biologically meaningful" if it contained at least one significantly enriched GO term. We restricted this analysis to *specific* GO terms, as generic and non-biologically informative GO terms often tend to show a statistically significant enrichment. Following a recommendation made by one of the referees, we defined GO terms that were four or less levels from the roots of the hierarchy defined in the gene ontology (version February 29, 2008) as generic, and discarded them from the subsequent analysis.

The results are shown in Table 3, which displays the number of biologically meaningful clusters (in Column 3) and the number of genes contained in them (Column 5). On the expression data, the proposed MFA-VBEM approach compares favorably with the competing methods and consistently shows the best performance. When combining gene expression data and TF binding profiles, MFA-VBEM consistently outperforms all other methods: a higher proportion of clusters is found to contain significantly enriched GO terms, and more genes are contained in these clusters. This is a demonstration of the robustness of MFA-VBEM in dealing with a certain violation of the distributional assumptions of the model; as a consequence of a thresholding operation applied to the experimentally obtained TF binding affinities, the TF binding profiles extracted from YeastTract [38] are binary rather than Gaussian distributed.

**Table 3:** Enrichment for GO terms in predicted gene clusters.

Data	Clusters	Biologically meaningful clusters	Genes	Genes in biologically meaningful clusters
Average linkage				

[35], E	48	10	3638	1483
[36], E	25	7	1993	1092
[35], E+B	30	8	3638	1148
[36], E+B	17	4	1993	703

K-means				

[35], E	48	18	3638	1847
[36], E	25	12	1993	987
[35], E+B	30	13	3638	1337
[36], E+B	17	9	1993	884

COSA				

[35], E	48	7	3638	1155
[36], E	25	8	1993	748
[35], E+B	30	10	3638	240
[36], E+B	17	4	1993	16

Plaid				

[35], E	48	19	3638	1812
[36], E	25	10	1993	770
[35], E+B	30	11	3638	626
[36], E+B	17	9	1993	636

MFA-VBEM				

[35], E	48	20	3638	2415
[36], E	25	16	1993	1278
[35], E+B	30	17	3638	2996
[36], E+B	17	14	1993	1645

The table shows the enrichment for known gene ontology (GO) terms in clusters predicted with different clustering algorithms from different data sets. Five clustering algorithms were compared: hierarchical agglomerative average linkage clustering, K-means, COSA [43], Plaid models [44], and the proposed MFA-VBEM scheme. The algorithms were applied to a combination of different microarray gene expression data. For the proposed MFA-VBEM algorithm, we additionally included the TF binding profiles of [34]. Clusters with significantly enriched GO terms (at the 5% significance level) are referred to as "biologically meaningful clusters". The number of genes in these clusters is shown in the rightmost column.

E: clustering based on gene expression data only; E+B: clusters obtained from both gene expression and TF binding data.

Interestingly, COSA shows a particularly poor performance on the combined gene expression and TF binding data. This can be explained as follows. The TF binding profiles extracted from YeastTract [38] are binary vectors, and some TFs bind to several genes. The affected genes will have identical (or very similar) binary profiles when restricted to the respective TFs. With its inherent tendency to cluster on subsets of attributes, COSA will group together genes that happen to have similar binary entries for a small number of TFs. This leads to the formation of many small clusters. These clusters are not necessarily biologically meaningful, since complementary information from the expression profiles and other TFs has effectively been discarded.

It is also interesting to observe that the inclusion of binding data occasionally deteriorates the performance of K-means and hierarchical agglomerative clustering. This deterioration is a consequence of the different nature of the TF binding and gene expression profiles. While the former are binary and hence nonnegative, the log gene expression ratios my vary in sign. This renders the approach of combining them in a monolithic block suboptimal, as coregulated genes may have anticorrelated expression profiles and positively correlated TF binding patterns. Avoiding this potential conflict by taking the modulus of the expression profiles is no solution, as the resulting information loss was found to lead to a deterioration of the clustering results. The proposed MFA-VBEM model, on the other hand, uses the extra flexibility that the model provides via the factor loading vector  and the factor mean vector  (see Figure 2) to overcome this problem. This suggests that MFA-VBEM provides the right degree of flexibility as a compromise between the rigidness of K-means and hierarchical agglomerative average linkage clustering, and the over-flexible subset selection of COSA. The consequence is an improvement in the biological plausibility of the inferred gene clusters, as seen from Table 3.

### 5.3. Regulatory Network Reconstruction

A topic of interest in computational systems biology is the reconstruction of transcriptional regulatory networks, and it is this question that most of the methods reviewed in the Introduction section ultimately aim to address. Note that in the current setting the regulatory network has the form of a bipartite graph between TFs and potentially regulated genes. The successful solution of the reconstruction task therefore requires us to infer for each TF the correct binding profile, that is, the set of genes that it potentially binds to and regulates. For the synthetic data of Section 4.1, this is a straightforward task as the true regulatory network is known. For real data, however, the true regulatory network is unknown, rendering the assessment more difficult. We approached this problem from two different angles: noise reduction and test-set performance. In the first assessment scheme, we trained (for descriptional convenience we use machine learning parlance, where the word "training" means inference of the posterior distribution of the model parameters, hyperparameters and latent variables from given data, the so-called training set) the different statistical models on noisy complete data—containing both gene expression profiles and TF binding affinities—and investigated whether the method succeeded in reducing the noise in the TF binding profiles, that is, whether it could predict a curated transcriptional regulatory network. In the second assessment scheme, the models were trained on 80% of the original data, and then evaluated on 20% of held-out test data, from which the binding profiles had been removed. We refer to these two network reconstructions tasks as network curation and network prediction, respectively. We compared the proposed MFA-VBEM scheme of Section 3 with the BFA Gibbs sampling approach of Sabatti and James [16] and with maximum likelihood FA. An overview of the methods compared in our study is shown in Table 1. Note that the PLS method of Boulesteix and Strimmer [22] was not applied to this task, as it provides no mechanism for inferring the TF-gene interaction strengths directly from gene expression data.

For reconstructing the transcriptional regulatory network with MFA-VBEM, we estimated the vector  of interaction strengths between gene  and all TFs from (24)

where  is a combined gene expression and TF binding profile included in the training set,  is given in (A.22), and  is obtained from (17) and (A.18). For the out-of-set prediction task, we computed the predicted interaction strengths from (25)

where  is a gene expression profile of a new gene not included in the training set, and  is computed by discarding from (A.22) all those terms that are related to the (nonexistent) TF binding profile. See the appendix for a derivation of (24) and (25). To obtain a regulatory network from the matrix of interaction strengths we choose a threshold and keep all those edges whose interaction strengths exceed this value. Note that by varying the threshold between the minimum and maximum interaction strength, we can obtain a receiver operating characteristic (ROC) curve when the true network is known.

We carried out maximum likelihood FA with the EM algorithm, using the software implementation of Ghahramani and Hinton [24], and a subsequent varimax rotation towards maximum sparsity of the loading matrix, as proposed in Pournara and Wernisch [18]. Since this approach does not make use of the TF binding data, the distinction between network curation and out-of-sample prediction is obsolete. Further details about the application of this scheme can be found in the appendix.

The network reconstruction with BFA-Gibbs was carried out as described in Sabatti and James [16]. For the out-of-sample network prediction, the Gibbs sampling scheme of Sabatti and James [16] was modified so as to set the TF activity profiles to the posterior mean obtained from the training set. This approach corresponds to running the Gibbs sampling algorithm of Sabatti and James [16] with the latent variables fixed, that is, one of the interleaved Gibbs steps is omitted. Again, further details and a justification of this scheme can be found in the appendix.

The practical application of BFA-Gibbs faces a computational hurdle. Within the Gibbs sampling procedure the vectors of binary latent variables ( in the notation of Pournara and Wernisch [18]) are sampled from a multinomial distribution whose parameters have to be computed for all possible configurations of  (Sabatti and James [16, (2)] or Pournara and Wernisch [18, (8)]). This is a combinatorial problem, and the computational costs increase exponentially with the number of non-zero entries in the prior probability matrix . For our simulations we used the software provided by Sabatti and James [16], which worked fine on the synthetic data of Section 4.1. However, the programs ran into memory overflow problems on the *S. cerevisiae* data when the number of nonzero entries in  was unrestricted. This computational complexity, which inherently impedes the application of BFA-Gibbs to complex postgenomic data sets, required us to artificially limit the number of nonzero entries in  to 11 connections per gene. Most of the *S. cerevisiae* genes were not affected by this intervention, as the number of TF binding connections reported in Teixeira et al. [38] is well below this threshold. However, for densely connected genes, TF binding connections had to be randomly discarded until the restriction was enforced. We note, though, that despite this pruning procedure, still 88% of the interactions reported in [38] were included in the prior probability matrix .

#### 5.3.1. Network Reconstruction for the Synthetic Data

**Figure 6:**
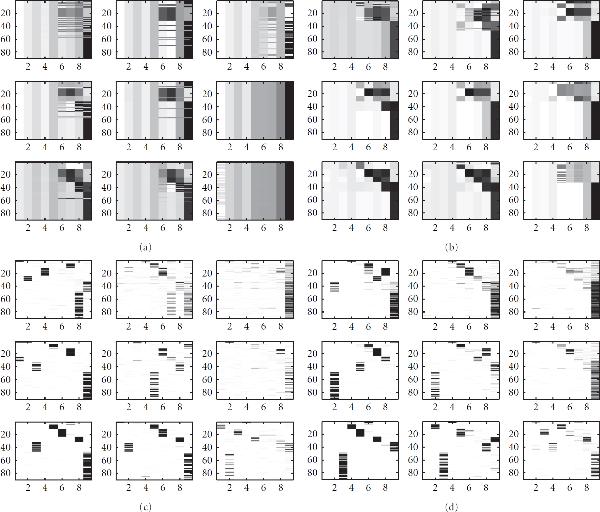
**TF-gene interactions reconstructed with MFA and BFA from the synthetic data**. The figure shows TF-gene interactions predicted with the proposed MFA-VBEM approach, according to (24), and the BFA-Gibbs method, according to (A.32), using the noisy synthetic gene expression profiles of Figure 3, and the synthetic TF binding data sets shown in Figures 4(c), 4(d). (a), (c) correspond to the noisy TF binding data shown in Figure 4(c). (b), (d) correspond to the less noisy TF binding data, shown in Figure 4(d). (a), (b) show the TF-gene interaction strengths predicted with the MFA-VBEM approach. (c), (d) show the corresponding results obtained with the BFA-Gibbs method. The grey shading indicates the predicted strength of the interactions, with white corresponding to the absence of an interaction, and black corresponding to the presence of an interaction. The horizontal axis in each graph represents the 9 TFs that are involved in the regulation of the 90 genes; the latter are represented by the vertical axis of each graph. In each panel, from top to bottom, the three rows correspond to gene expression profile lengths of 10, 20 and 40. The three columns correspond to the three noise levels of the gene expression profiles. From left to right:  and . See Section 4.1 for further details.

**Figure 7:**
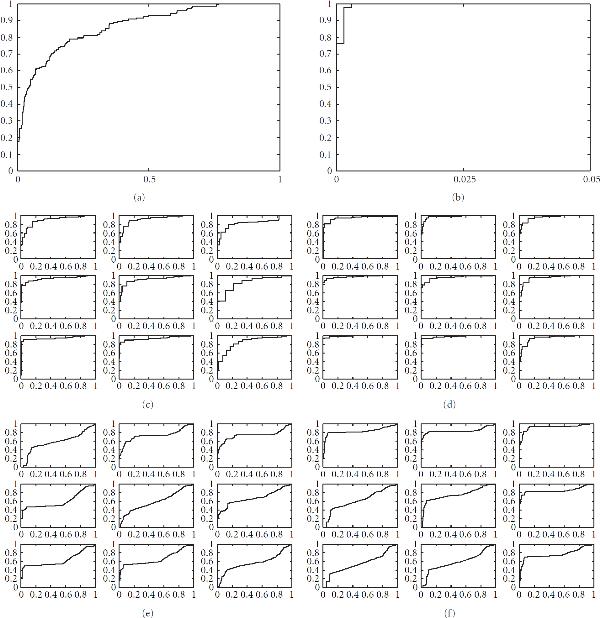
**ROC curves of TF-gene regulatory network reconstruction for the synthetic data with MFA and BFA**. This figure shows various receiver operating characteristic (ROC) curves, where the numbers of predicted true positive interactions (vertical axis) are plotted against the numbers of false positive interactions (horizontal axis). Larger areas under the curve (AUC) indicate a better reconstruction accuracy. (a), (b) show the ROC curves obtained from TF binding data alone, without including gene expression profiles. (a) corresponds to the noisy TF binding data shown in Figure 4(c). (b) corresponds to the less noisy TF binding data, shown in Figure 4(d). (c), (d) each composed of 9 graphs, show the predictions obtained with MFA-VBEM from both noisy TF binding and gene expression profiles. (e), (f) also composed of 9 graphs each, show the results obtained with BFA-Gibbs on the same data. The arrangement of the graphs is the same as in Figure 6. The results suggest that MFA-VBEM systematically outperforms BFA-Gibbs. They also suggest that for noisy TF binding data (c), (e), the inclusion of gene expression profiles and the application of MFA-VBEM leads to an improvement in the TF-gene regulatory network reconstruction.

Figures 6 and 7 show the predicted TF-gene interactions for the synthetic data of Section 4.1. The synthetic gene expression profiles are shown in Figures 3(b), 3(c), 3(d). The (noisy) TF binding profiles are shown in Figures 4(c), 4(d). Figure 6 shows the TF-gene interactions predicted with the proposed MFA-VBEM method, according to (24), and the BFA-Gibbs method of Sabatti and James [16], using (A.32). Figure 7 shows the corresponding receiver operating characteristic (ROC) curves. For the noisy TF-gene binding data (left panels in Figures 6 and 7), the integration of gene expression profiles and the application of MFA-VBEM leads to an improvement in the reconstruction of the TF-gene interactions. This improvement is particularly noticeable for the longer gene expression profiles () and the lower noise levels (). For the low noise level on the TF binding profiles (right panels in Figures 6 and 7), there is no room for improvement. It is reassuring that the integration of noisy gene expression profiles with the MFA-VBEM method results only in a small deterioration, while the deterioration with BFA-Gibbs is much more substantial. A comparison between the top and bottom panels of Figure 6, and between the centre and bottom panels of Figure 7 suggests that MFA-VBEM significantly outperforms BFA-Gibbs. In particular, it is seen that BFA-Gibbs fails to predict the existence of TF complexes. Most genes are predicted to be regulated by a single TF, whereas in fact genes are regulated by TF complexes consisting of up to three TFs (as correctly predicted with MFA-VBEM).

Interestingly, for the low noise in the TF binding data, from which the prior connectivity matrix  of BFA-Gibbs is derived, the performance of BFA-Gibbs is relatively better when the gene expression profiles are noisy (the right column in the bottom right panel of Figure 7), or the gene expression profiles are short (top row in the bottom right panel of Figure 7). We have obtained similar results on the reconstruction of TF module activity profiles (Table 2). With larger, less noisy data sets, the Gibbs sampler can be easily trapped in some local optimum. This is partly related to MCMC sampling problems in general; compare with Figures 6 and 7 in Grzegorczyk and Husmeier [49]. More substantially, this is related to mixing problems inherent in Gibbs sampling. There are  possibilities to assign a TF to the six groups of coexpressed genes in Figure 4, corresponding to  modes in the posterior probability landscape. A study by Jasra et al. [50] has found that in such a scenario Gibbs sampling faces intrinsic mixing problems and tends to get trapped on a single mode. Note that both problems are avoided by the proposed MFA-VBEM scheme. First, by information sharing between TFs in the same module, MFA effectively constitutes a more parsimonious model than BFA, thereby reducing the complexity of the inference problem. Second, convergence problems are effectively addressed with the birth-death moves in a similar way as discussed in Ueda et al. [32].

A comparison of the original TF-binding data in Figure 4 and the predicted TF-gene interaction profiles in Figure 6 clearly demonstrates the efficiency of the network curation and noise reduction affected with MFA-VBEM. Note that the improved reconstruction accuracy is a consequence of the systematic integration of gene expression data into the modelling and inference process, and the nature of the MFA model. The latter allows for the fact that TFs act in modules and are non-independent, and that TFs in the same module show similar interaction patterns with downstream regulated genes. This leads to greater robustness of inference and reduced susceptibility to overfitting.

#### 5.3.2. Network Reconstruction for the Yeast Data

**Figure 8:**
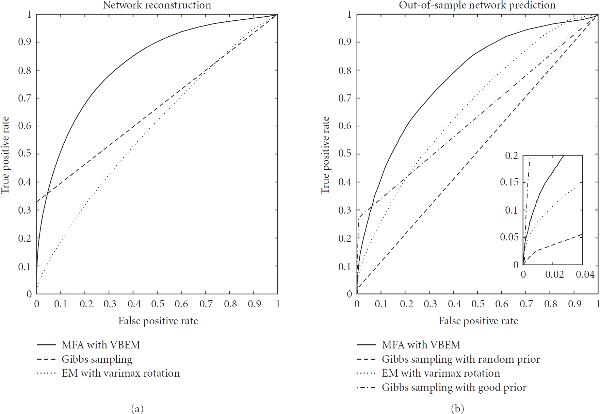
**TF regulatory network reconstruction for yeast**. Receiver operating characteristic (ROC) curves obtained for *S. cerevisiae* with three different methods: (1) solid line: the proposed MFA-VBEM method, based on the work of [23], and extended as described in Section 3; (2) dashed line: the Bayesian FA model with Gibbs sampling, as proposed in Sabatti and James [16]; and (3) dotted line: maximum likelihood FA with the EM algorithm of Ghahramani and Hinton [24] and a subsequent varimax rotation [39] of the loading matrix towards maximum sparsity, as proposed in Pournara and Wernisch [18]. (a) The performance on a noisy training set, where 10% false positive interactions had been randomly added to the TF binding profiles from the literature [38], while the computation of the ROC curves was based on the un-perturbed literature data (network curation task). (b) The out-of-sample performance on an independent test set containing genes not used for training (network prediction). Note that in the latter case the Gibbs sampling approach was run twice, with two different prior matrices : a random prior, where for each gene 11 randomly chosen elements in the matrix were nonzero (dashed line); and a "good" prior, where the nonzero elements in  were chosen according to Teixeira et al. [38] subject to the maximum connectivity constraint described in the text (dash-dotted line).

Table 1 provides an overview of how the TF binding strengths are predicted with the different methods compared in our study. From these scores we can obtain the prediction of a specific TF regulatory network by discarding all interactions below a given threshold. Taking the binding profiles reported in Teixeira et al. [38] as a gold standard, we obtain for the chosen threshold a ratio of true positive (TF) and false positive (FP) regulatory interactions. Rather than selecting an arbitrary value for the threshold, we can plot for all possible thresholds the TP ratios against the FP ratios. This leads to the receiver operating characteristic (ROC) curves of Figure 8, where larger areas under the curve (AUC) indicate a better reconstruction accuracy.

For the network curation task, 10% false-positive interactions were added to the TF binding data of Teixeira et al. [38]. All three models were trained using the complete data set, including both gene expression and (noisy) TF binding profiles. We then assessed the predicted binding profiles by taking the associations reported in Teixeira et al. [38] as the true gold standard. The resulting ROC curves are shown in the left panel of Figure 8.

BFA with Gibbs sampling recovered a very accurate but sparse connectivity matrix. Most of the predicted connections were correct according to the chosen criterion (agreement with Teixeira et al. [38]). However, only about 30% of the TF binding connections reported in Teixeira et al. [38] were recovered, and 20% of the genes were predicted to be not connected to any TF. Additionally, most genes were predicted to be connected to at most one TF, which suggests that BFA-Gibbs does not capture any effects related to TF complex formation and cooperativity between TFs. The proposed MFA-VBEM approach avoided this problem by predicting many genes to be connected to more than one TF. For very low FP rates MFA-VBEM obtained lower TP rates than BFA-Gibbs. However, its area under the ROC curve (AUC score) is substantially higher than that of BFA-Gibbs (0.82 versus 0.66), suggesting that the overall prediction performance has improved. The performance of maximum likelihood factor analysis (FA) was much poorer than that of the other two methods, and the corresponding ROC curve was only marginally better than the expected performance of a random predictor. Recall that FA as opposed to the other two models only uses the gene expression data but not the TF binding profiles. The poor performance of FA thus suggests that the TF regulatory network cannot be reliably reconstructed on the basis of gene expression data alone, and that the varimax rotation of the loading matrix towards maximum sparsity, as suggested in Pournara and Wernisch [18], is no substitute for the explicit inclusion of TF binding information.

For the network prediction task, we trained the models on only 80% of the *S. cerevisiae* genes, and used an independent test set containing a randomly chosen subset of 20% of the genes to estimate the out-of-sample network prediction accuracy. Note that for the genes in the test set, only the expression profiles were made available, while the corresponding TF binding connections were held back. The task was to predict these TF binding connections from the gene expression data, using the (average) TF activity profiles inferred from the training set. A more comprehensive description of the evaluation is provided in the appendix.

The results are shown in the right panel of Figure 8. This figure contains two ROC curves for BFA-Gibbs. The proper evaluation of the out-of-sample network prediction accuracy according to equation (A.35) requires an uninformative prior connectivity matrix  for the genes in the test set, in which all the elements are set to . However, the combinatorial complexity problem discussed above requires a restriction on the number of non-zero entries per genes. We randomly selected a set of 11 non-zero entries per gene. This leads to the ROC curve shown by a dashed line in the right panel of Figure 8, which is hardly better than the expected ROC curve of a random predictor. This poor performance is not surprising, because BFA-Gibbs cannot recover false negative interactions, as discussed in Sabatti and James [16]. As an alternative test, we selected the true TF binding interactions, as reported in Teixeira et al. [38], subject to the constraint of not allowing more than 11 non-zero entries  per gene. The corresponding ROC curve is shown by the dash-dotted line in the right panel of Figure 8, which outperforms all other methods for low FP ratios. Note, though, that this approach violates the out-of-sample paradigm, in that it makes use of TF binding information that should have been held back for evaluation. Interestingly, even with this methodological violation, BFA-Gibbs is still outperformed by the proposed MFA-VBEM approach in terms of the global network reconstruction accuracy, as indicated by the overall AUC score. MFA-VBEM also significantly outperforms maximum likelihood FA (dotted graph in the right panel of Figure 8(b)). (It might seem peculiar that the out-of-sample performance of FA, as shown in Figure 8(b), is better than the training set performance, depicted on the left. This is a consequence of the global assignment of predicted TF binding profiles to true binding profiles with the Hungarian algorithm, as described in Section A.3, which works more efficiently on smaller data sets. As discussed before, this procedure uses information that should have been withheld, giving FA an unfair advantage over the other methods.) Consistently achieving higher TP ratios across the whole spectrum of FP ratios.

While the previous study has pointed to a performance improvement of MFA-VBEM over BFA-Gibbs, this improvement is a combination of two effects: the actual model performance, and the computational complexity. In order to focus on the first effect and distinguish it from the latter, we repeated the analysis on the same data in a slightly different manner. Recall that the proper evaluation of the out-of-sample network prediction accuracy according to (A.35) requires an uninformative prior connectivity matrix  for the genes in the test set, and that the combinatorial complexity problem discussed above requires a restriction on the number of non-zero entries per gene. We therefore randomly selected 2000 *S. cerevisiae* genes, then sorted the TFs according to the numbers of connections between them and the selected genes. The most densely connected 12 TFs were chosen. Then all 5464 genes were sorted according to the numbers of their connections to the chosen TFs, and the most densely connected 2000 genes were chosen. These sorting steps were iterated until convergence. We thus obtained a 12 TFs by 2000 genes connectivity matrix with dense connections for evaluating the different network reconstruction methods. This procedure, and the reduction in the number of TFs, allowed the application of BFA-Gibbs with an uninformative prior connectivity matrix, and hence ensured a fair comparison with the proposed MFA-VBEM method.

**Figure 9:**
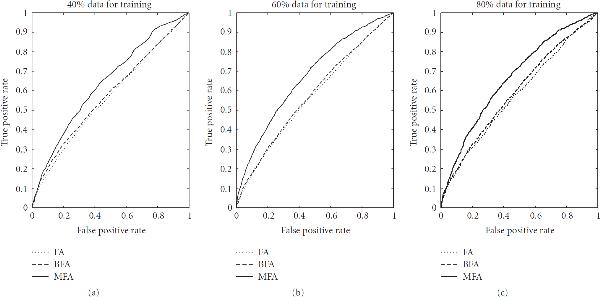
**Out-of-sample TF regulatory network reconstruction for yeast**. Receiver operating characteristic (ROC) curves obtained for *S. cerevisiae* with three different methods: (1) solid line: the proposed MFA-VBEM method, based on the work of Beal [23], and extended as described in Section 3; (2) dashed line: the Bayesian FA model with Gibbs sampling, as proposed in Sabatti and James [16]; and (3) dotted line: maximum likelihood FA with the EM algorithm of Ghahramani and Hinton [24] and a subsequent varimax rotation [39] of the loading matrix towards maximum sparsity, as proposed in Pournara and Wernisch [18]. The subfigures show the out-of-sample performance on an independent test set containing genes not used for training (network prediction). From left to right, the models were trained using 40%, 60% and 80% of data.

For the network prediction task, we trained the models on randomly selected 40%, 60% or 80% of the *S. cerevisiae* genes, and used an independent test set containing the remaining 60%, 40% or 20% of the genes to estimate the out-of-sample network prediction accuracy. As before, for the genes in the test set only the expression profiles were made available, while the corresponding TF binding connections were held back. The task was to predict these TF binding connections from the gene expression data, using the (average) TF activity profiles inferred from the training set. The results are shown in the subfigures of Figure 9. It can be seen in all three cases that MFA with VBEM clearly outperforms both the BFA and FA methods, and that the performance slightly increases with increasing training set size. The corresponding AUC values are 0.64, 0.67 and 0.67. 

**Figure 10:**
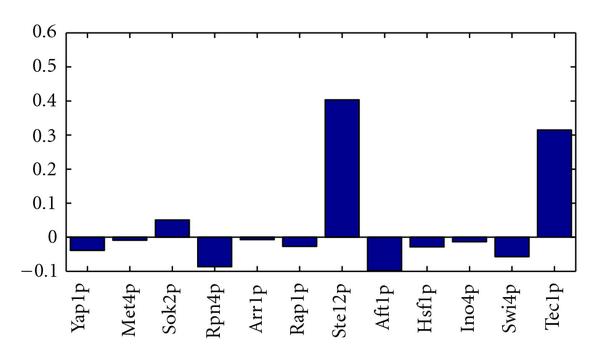
**Composition of one of the TF complexes in yeast**. The figure shows the composition of one of the TF modules () found with MFA-VBEM for the yeast data. The figure shows a plot of , plotted on the vertical axis against the 12 TFs involved in the study. As explained in the caption of Figure 2,  indicates the composition of the th TF module. It is clearly seen that this TF module is dominated by two TFs, Ste12 and Tec1, and thereby corresponds to a well-established module reported in the literature [51].

Of particular interest is that among the 12 TFs of the yeast set, there is a well established TF complex (module) composed of TFs Ste12 and Tec1 [51]. This TF module is clearly recognised by one of the components of our MFA-VBEM model, as shown in Figure 10.

The measured TF-gene binding patterns of these two TFs show a modest correlation (correlation coefficient = 0.60). When MFA-VBEM is applied to the network reconstruction task by integrating gene expression profiles, the predicted binding patterns of the two TFs involved in the complex show an increased correlation (correlation coefficient = 0.74). However, the cooperation of TFs was not detected by the BFA or the FA methods. Here, the corresponding correlation coefficients between the TF binding patterns predicted with BFA and FA are low, 0.15 and 0.14, respectively. Hence, BFA and FA fail to identify this TF complex.

## 6. Conclusion

We have investigated the application of Bayesian mixtures of factor analyzers (MFA-VBEM) to modelling transcriptional regulation in cells. Like recent approaches based on Bayesian factor analysis applied to the same problem [16, 17], MFA-VBEM allows for the fact that TFs are often subject to post-translational modifications and that their true activities are therefore usually unknown. A shortcoming of Bayesian factor analysis is the fact that it ignores interactions between TFs. This limitation is addressed by our approach: different from Bayesian factor analysis, the mixture of factor analyzers approach allows for the fact that transcription factors co-operate as a functional complex in regulating gene expression, which is particularly common in higher eukaryotes. Our approach systematically integrates gene expression data with TF binding data. As opposed to the partial least squares (PLS) approach of Boulesteix and Strimmer [22], MFA-VBEM is a probabilistic model that allows for the noise inherent in the TF binding data. This addresses a major shortcoming of the PLS approach, where the inability to deal with measurement errors has been found to adversely affect the activity profile reconstruction accuracy. The better performance of the MFA-VBEM method over the Bayesian factor analysis approaches is presumably a consequence of the more parsimonious model that results when allowing for the fact that TFs are non-independent. Take, for instance, a complex of 3 TFs that regulates 20 genes, as in Figure 4. MFA-VBEM can effectively model this with 23 parameters: 20 regulatory interaction strengths between the TF module and the regulated genes, and 3 membership indicators that assign the TFs to the respective module. A method based on the standard FA approach, like the one proposed by Sabatti and James [16], needs  parameters, corresponding to the interactions between each of the individual TFs and the regulated genes. There is nothing in the FA approach that would inform the model a priori that once a group of TFs are found to form a module, their interaction patterns with the regulated genes should be the same. Instead, these interaction strengths have to be learned separately for each TF. This leads to a less parsimonious and partially redundant model, which is less robust and more susceptible to over-fitting.

We have evaluated the proposed MFA-VBEM on three performance criteria: transcriptional activity profile reconstruction, gene clustering, and regulatory network inference. Using a synthetic data set, we found that MFA-VBEM reconstructed the hidden activity profiles of the TF complexes more accurately than PLS [22] and Bayesian factor analysis with Gibbs sampling [16]. Using gene expression profiles and TF binding profiles for *S. cerevisiae*, MFA-VBEM found biologically more plausible gene clusters than K-means, hierarchical agglomerative average linkage clustering and COSA [43], as indicated by the increased enrichment for known gene ontology terms. For the regulatory network reconstruction task, MFA-VBEM outperformed Bayesian and non-Bayesian factor analysis models on gene expression and TF binding profiles from both *S. cerevisiae* and a synthetic simulation. The better performance over the Gibbs sampling approach of Sabatti and James [16] on *S. cerevisiae* was partly a consequence of the computational complexity of the latter approach; this highlights the practical advantage of the proposed scheme in scaling up to complex postgenomic data sets.

We have pursued a variational approach to Bayesian inferences, by which a lower bound on the marginal likelihood is obtained and used for model selection. This allows us to estimate the number  of active transcriptional modules regulating the genes, and select the number most supported by the data. A straightforward extension would be to make the number of active transcriptional modules a random variable itself and estimate its posterior distribution. The question, then, is which prior distribution to place on it. The potential number of active transcriptional modules is large, owing to the combinatorial explosion inherent in TF cooperation. Moreover, biological regulatory networks are known to be scale-free [52], meaning that a few TF modules potentially regulate a large number of genes. These two properties suggest that a Dirichlet process prior (also called Chinese restaurant process) would provide the appropriate modelling framework [53]. This non-parametric approach to Bayesian modelling has become popular in the machine learning community, and has recently been applied to computational biology in the context of haplotype modelling [54]. The application of these ideas to the problem of transcriptional regulation, and the method discussed in the present paper in particular, will provide an interesting avenue for future research.

## Appendix

### A. 

#### A.1. Variational Bayesian Expectation Maximization

This section provides a concise review of variational inference. For a more comprehensive tutorial, we refer the reader to Bishop [42]. Consider the simple Bayesian FA model with latent variables  and parameters , where the latter are treated as random variables for which some prior distribution is defined. The objective of Bayesian inference is to infer the posterior distribution  from the data  for some model , and to decide on the best model  on the basis of the marginal likelihood . In the context of the FA model of (A.3) and (A.25), model selection means deciding on the dimension of the latent space, dim(). In the context of mixtures of FA models, discussed in Section 3, the model selection task is to decide on the number of components in the mixture. Unfortunately, neither  nor  can be computed in closed form. The objective of variational inference is to approximate both on the basis of an analytically tractable model distribution . Define (A.1)

It is easy to show that  can be decomposed into the following form: (A.2)

which is the difference of the log marginal likelihood and the Kullback-Leibler divergence between the model distribution  and the unknown true posterior distribution : (A.3)

From information theory it is known that the Kullback-Leibler divergence, which is a measure of the difference between two distributions, is non-negative; see, for instance, Papoulis [48]. This implies that  is a lower bound on the marginal likelihood , with a difference given by the the Kullback-Leibler divergence . The objective of variational Bayesian inference is to numerically maximize . This gives the best approximation to the true posterior distribution from the functional family , while simultaneously  gives the best possible approximation to the marginal likelihood.

To apply the concept of variational learning to Bayesian FA, the model distribution is assumed to factorize into separate contributions from the parameters and latent variables: . The variational learning algorithm then iteratively maximizes the functional  with respect to the free distributions  and . Given a fixed distribution of the parameters ,  is maximized with respect to  by setting to zero the following functional derivative: (A.4)

where  is a Lagrange multiplier resulting from the normalization constraint. Equation (A.4) has the closed-form solution: (A.5)

where  denotes a normalization constant. Likewise, given a fixed distribution of the latent variables ,  is maximized with respect to  by setting to zero the following functional derivative: (A.6)

which has the closed form solution (A.7)

Again,  is a normalization constant. For a derivation of these results, see, for example, Nielsen [25] and Beal [23]. For distributions of the exponential family, which includes FA, (A.5) and (A.7) have a closed-form solution, as shown, for example, in Beal [23]. In analogy to the Expectation Maximization (EM) algorithm, the variational learning algorithm follows an iterative adaptation procedure including the following two steps: 

(i) variational E-step: given the distribution of the parameters , where  indicates the iteration number, obtain a new distribution of the latent variables  by application of (A.5). 

(ii) variational M-step: given the distribution of the latent variables , obtain a new distribution of the parameters  by application of (A.7). 

This procedure, called the Variational Bayesian Expectation Maximization (VBEM) algorithm, is repeated until a stationary point of  is reached.

#### A.2. The VBEM Algorithm Applied to the MFA Model

We briefly describe the VBEM algorithm for the MFA model discussed in Section 3, which is derived from (22) by applying the variational calculus outlined in Section A.1. A complete derivation of the update equations can be found in Beal [23]. We here present a straightforward modification of these equations for variational Bayesian inference in the model presented in Section 3. Recall that the dependence structure between the (hyper-) parameters and latent variables is depicted in Figure 2, and the factorization of the variational distribution is described in the text below (21). The variational posterior distribution of the mixture components  is a Dirichlet distribution: (A.8)

in which (A.9)

 was defined above equation (11), and  is taken from (A.22). The precision parameters  are gamma distributed: (A.10)

where  and  are the hyperparameters of the prior distribution in (20), and  denotes an expectation value with respect to the variational distribution , computed from (A.11) and (A.12). The variational posterior distribution of the centres  and loading vectors , concatenated into the -by-2 matrix (A.11)

is a multivariate Gaussian distribution: (A.12)

in which  denotes the column vector corresponding to the th row of , and the variational posterior parameters are defined as follows: (A.13)

with (A.14)(A.15)(A.16)(A.17)(A.18)

where  denotes an expectation value with respect to , obtained from (A.10),  denotes an expectation value with respect to , obtained from (A.19),  is the th component of  is the th component of  is the th component of , and the index  is related to a time point or experimental condition for which microarray and TF binding data have been obtained.

The variational posterior for the hidden factor , conditioned on the indicator variable , is given by (A.19)

in which (A.20)(A.21)

where  denotes an expectation value with respect to , obtained from (A.12).

The variational posterior distribution for the indicator variables  is given by (A.22)

Here,  denotes an expectation value with respect to the distributions  and , obtained from (A.12) and (A.19),  is a normalization factor to ensure that  was defined in (A.11),  was defined in (A.9),  is the trace operator, and  is the digamma function, defined as (A.23)

Recall from Section 3 and Figure 2 that the covariance matrix of the noise  is not treated as a random variable, but as a parameter (it has no prior distribution). For estimating , the derivative of  in equation (22) with respect to  is set to zero, which leads to the following update equation: (A.24)

Here,  denotes an expectation value with respect to the distributions  and , obtained from the previous update steps in equations (A.12), (A.22) and (A.19), and  was defined in (A.11).

The hyperparameters  and  are obtained in the same way, leading to the following expressions: (A.25)

where , and  denotes an expectation value with respect to the distribution , which is obtained from (A.11) and (A.12). The remaining hyperparameters were fixed at , corresponding to fairly vague prior distributions. Each update equation is guaranteed to increase  of (22), and the update steps are repeated in an iterative procedure until a stationary point of  is reached. This update procedure involves birth and death moves to explore the model space and find the optimal model complexity , as described in Beal [23]. Note that these birth and death moves also help avoid local maxima in  of (22), in a similar manner as discussed in Ueda et al. [32]. A MATLAB implementation of this method has been made available by Beal [23].

#### A.3. Details on the Regulatory Network Reconstruction

Network Reconstruction with The Proposed Mfa-Vbem Scheme

Mathematically, the TF binding profile predicted from the training data  is given by (A.26)

where  is the TF binding profile of gene , and  is the training set, which contains (noisy) expression data  and (noisy) binding data  for gene : . The posterior probability  is given by a marginalization over the latent variables  and , the model parameters  and , and the hyperparameters ; see Figure 2. Note that the other hyperparameters, represented by square boxes in Figure 2, are fixed, optimized so as to maximize  in (22) (note that this corresponds to a maximum likelihood type II estimation, with the marginal likelihood approximated by its lower bound ): (A.27)

The integral in (A.27) is analytically intractable. In an MCMC setting, it would be approximated by a sum over parameters, hyperparameters and latent variables sampled from a Markov chain. In the variational approach, the posterior distribution is approximated by (A.28)

where expressions for the variational distributions  can be found in Section A.2. Inserting these expressions into (A.28), and making use of (A.27) and (14), we obtain: (A.29)

where  is given in (A.22),  is given in (A.18),  is given in (A.17), and  is given in (A.21). The curated binding profile  of gene , corresponding to (A.26), is then trivially obtained from  by discarding the expression profile  in . Note that (A.29) consists of two terms. The first term, , describes the potential binding of TF modules to the promoters of the regulated genes. This is the generic regulatory network that we want to predict, mediated via regulated elements in the gene upstream sequences. The second term, , describes the perturbations and transient modifications of the interactions that are specific to the experimental conditions for which the training data were obtained. This term allows for the fact that a potential binding site might not be accessible to a TF in a certain condition, and that the TF binding affinities vary with changing external conditions.

For the out-of-sample network prediction, we want to compute the conditional expectation value of the binding profile (A.30)

from an expression profile , where  is not included in the training set . On the assumption that the training set is sufficiently large, we can approximate the posterior distribution by , as it is not going to be noticeably changed by the inclusion of a single additional observation. The variational approximation then leads to (A.31)

where  is in principle obtained by application of (A.22) to obtain , and marginalization over : . Since the derivation of  is involved, we approximate  by discarding from (A.22) all those terms that are related to the TF binding profiles; the trace operator in (A.22) thus extends over contributions from the gene expression data  only. Note that this approximation corresponds to the imputation  with . Our results suggests that this approximation works sufficiently well in practice.

Having described how to approach the tasks of network curation and prediction with the proposed MFA-VBEM model, we will now briefly outline how to address these problems with the factor analysis models of Sabatti and James [16] and Ghahramani and Hinton [24].

Network Reconstruction with Maximum Likelihood Factor Analysis

Recall the definition of the FA model in (A.3) and (A.25). The EM approach proposed in Ghahramani and Hinton [24] consists of iterative adaptation steps for the latent factors  (representing TF activity profiles), the parameters  (representing regulatory connection strengths), and the noise parameters . To solve the identifiability problem inherent in FA we follow Pournara and Wernisch [18] and minimize the number of non-zero entries in the connection strength matrix  with a varimax rotation [39]; this procedure incorporates our prior knowledge that biological regulatory networks are usually sparsely connected. Maximum likelihood FA works solely with the gene expression data and does not incorporate explicit information about TF binding profiles. Consequently, the distinction between network curation and network prediction is not essential, and is solely made for comparison with the competing models. In the "curation" task, the connectivity matrix  inferred from the training set in the way described above is used as the prediction of the transcriptional regulatory network. In the "prediction" task, the TF activity profiles  obtained from the training data are kept fixed, and the TF regulatory network, represented by , is estimated for a set of independent genes (the test data). This procedure, which is straightforwardly implemented by skipping the E-step in the EM algorithm, indirectly tests how accurately the TF activity profiles  have been reconstructed.

For the practical application, we applied the EM algorithm as reported in Ghahramani and Hinton [24], using the MATLAB programs provided by the authors. Each EM optimization was repeated five times from different random initializations, and the result with the highest likelihood was kept for further analysis. Since standard FA does not use any information from the TF binding profiles, the hidden factors cannot be immediately associated with known TFs. In order to evaluate how accurately the estimated loading matrix  predicts the transcriptional regulatory network, we mapped each hidden factor to the closest TF. This was effected by an application of the Hungarian algorithm (The Hungarian algorithm is a combinatorial optimization algorithm. The assignment problem is represented by a cost matrix, where each matrix element represents the cost of assigning a predicted TF profile to a real TF binding profile. The algorithm solves the assignment problem in polynomial time, finding the minimum edge weight matching for the bipartite graphs.) [55] to assign the hidden factors to the known TFs in such a way that the global Euclidean distance between the corresponding rows in  and the TF binding profiles reported in Teixeira et al. [38] was minimized. Note that this procedure requires the TF binding profiles to be already known beforehand, which would not be the case in practical applications, and that it therefore gives maximum likelihood FA a slight advantage over the other methods used in the comparison.

Network Reconstruction with Bayesian Factor Analysis and Gibbs Sampling

The Bayesian factor analysis model of Sabatti and James [16] was discussed in Section 2. Prior knowledge about the transcription factor binding profiles is incorporated via the mixture prior [27] of (7)–(9). The network curation task corresponds to the estimation of (A.32)

which is straightforwardly affected with the Gibbs sampling procedure described in Sabatti and James [16]. Recall that the data  correspond to the gene expression profiles, while the TF binding profiles are incorporated via the prior knowledge matrix . The task of network prediction can be formulated as follows: use the model obtained from the gene expression profiles  and TF binding profiles  to predict a regulatory network for a set of new genes with expression profiles , and a new prior matrix . Mathematically, this notion can be interpreted in two ways: either using the entire posterior distribution of TF activities from the combined training and test set, or using the posterior mean TF activity profiles from the training set as a plug-in estimator on the test set. The first approach corresponds to (A.33)

The practical application would require us to re-run the Gibbs sampling algorithm of Sabatti and James [16] on an augmented gene expression set . The second interpretation corresponds to inferring the posterior average TF activity profiles from the training set (A.34)

When expression profiles of new genes  with TF binding profiles  are obtained, the posterior average TF activity profiles are used to predict the regulatory network connections via (A.35)

This approach corresponds to running the Gibbs sampling algorithm of Sabatti and James [16] with the latent variables  fixed, that is, one of the interleaved Gibbs steps can be omitted. The second approach is computationally cheaper than the first and also appears more in line with the concept that a held-out test set should not be used for parameter inference. It was therefore adopted in our study.

## References

[B1] Bussemaker HJ Li H Siggia ED Regulatory element detection using correlation with expression Nature Genetics 2001 27 2 167 171 10.1038/84792 11175784

[B2] Conlon EM Liu XS Lieb JD Liu JS Integrating regulatory motif discovery and genome-wide expression analysis Proceedings of the National Academy of Sciences of the United States of America 2003 100 6 3339 3344 10.1073/pnas.0630591100 12626739 PMC152294

[B3] Beer MA Tavazoie S Predicting gene expression from sequence Cell 2004 117 2 185 198 10.1016/S0092-8674(04)00304-6 15084257

[B4] Phuong TM Lee D Lee KH Regression trees for regulatory element identification Bioinformatics 2004 20 5 750 757 10.1093/bioinformatics/btg480 14751992

[B5] Segal E Yelensky R Koller D Genome-wide discovery of transcriptional modules from DNA sequence and gene expression Bioinformatics 2003 19 supplement 1 i273 i282 10.1093/bioinformatics/btg1038 12855470

[B6] Middendorf M Kundaje A Wiggins C Freund Y Leslie C Predicting genetic regulatory response using classification Bioinformatics 2004 20 supplement 1 i232 i240 10.1093/bioinformatics/bth923 15262804

[B7] Middendorf M Kundaje A Shah M Freund Y Wiggins CH Leslie C Motif discovery through predictive modeling of gene regulation Proceedings of the 9th Annual International Conference on Research in Computational Molecular Biology (RECOMB '05), Cambridge, Mass, USA, May 2005 538 552

[B8] Ruan J Zhang W A bi-dimensional regression tree approach to the modeling of gene expression regulation Bioinformatics 2006 22 3 332 340 10.1093/bioinformatics/bti792 16303796

[B9] Raychaudhuri S Stuart JM Altman RB Principal components analysis to summarize microarray experiments: application to sporulation time series Pacific Symposium on Biocomputing 2000 5 455 466 10.1142/9789814447331_0043 PMC2669932 10902193

[B10] Liebermeister W Linear modes of gene expression determined by independent coponent analysis Bioinformatics 2002 18 1 51 60 10.1093/bioinformatics/18.1.51 11836211

[B11] Liao JC Boscolo R Yang Y-L Tran LM Sabatti C Roychowdhury VP Network component analysis: reconstruction of regulatory signals in biological systems Proceedings of the National Academy of Sciences of the United States of America 2003 100 26 15522 15527 10.1073/pnas.2136632100 14673099 PMC307600

[B12] Kao KC Yang Y-L Boscolo R Sabatti C Roychowdhury V Liao JC Transcriptome-based determination of multiple transcription regulator activities in *Escherichia coli* by using network component analysis Proceedings of the National Academy of Sciences of the United States of America 2004 101 2 641 646 10.1073/pnas.0305287101 14694202 PMC327201

[B13] Harbison CT Gordon DB Lee TI Transcriptional regulatory code of a eukaryotic genome Nature 2004 430 7004 99 104 10.1038/nature02800 PMC3006441 15343339

[B14] Bailey TL Elkan C Fitting a mixture model by expectation maximization to discover motifs in biopolymers Proceedings of the 2nd International Conference on Intelligent Systems for Molecular Biology (ISMB '94), Stanford, Calif, USA, August 1994 28 36 7584402

[B15] Hughes JD Estep PW Tavazoie S Church GM Computational identification of *cis*-regulatory elements associated with groups of functionally related genes in *Saccharomyces cerevisiae* Journal of Molecular Biology 2000 296 5 1205 1214 10.1006/jmbi.2000.3519 10698627

[B16] Sabatti C James GM Bayesian sparse hidden components analysis for transcription regulation networks Bioinformatics 2006 22 6 739 746 10.1093/bioinformatics/btk017 16368767

[B17] Sanguinetti G Lawrence ND Rattray M Probabilistic inference of transcription factor concentrations and gene-specific regulatory activities Bioinformatics 2006 22 22 2775 2781 10.1093/bioinformatics/btl473 16966362

[B18] Pournara I Wernisch L Factor analysis for gene regulatory networks and transcription factor activity profiles BMC Bioinformatics 2007 8 1 20 article 61 10.1186/1471-2105-8-61 17319944 PMC1821042

[B19] Shi Y Simon I Mitchell T Bar-Joseph Z Vingron M, Wong L A combined expression-interaction model for inferring the temporal activity of transcription factors Proceedings of the 12th Annual International Conference on Research in Computational Molecular Biology (RECOMB '08), Lecture Notes in Computer Science, Singapore, March-April 2008 4955 Springer 82 97

[B20] Reményi A Schöler HR Wilmanns M Combinatorial control of gene expression Nature Structural & Molecular Biology 2004 11 9 812 815 10.1038/nsmb820 15332082

[B21] Yu X Lin J Zack DJ Qian J Identification of tissue-specific *cis*-regulatory modules based on interactions between transcription factors BMC Bioinformatics 2007 8 1 13 article 437 10.1186/1471-2105-8-1 17996093 PMC2194798

[B22] Boulesteix A-L Strimmer K Predicting transcription factor activities from combined analysis of microarray and ChIP data: a partial least squares approach Theoretical Biology & Medical Modelling 2005 2 1 12 article 23 10.1186/1742-4682-2-1 15978125 PMC1182396

[B23] Beal MJ Variational algorithms for approximate Bayesian inference, Ph.D. thesis 2003 Gatsby Computational Neuroscience Unit, University College London, London, UK

[B24] Ghahramani Z Hinton GE The EM algorithm for mixtures of factor analyzers 1996 CRG-TR-96-1 Department of Computer Science, University of Toronto, Toronto, Canada

[B25] Nielsen FB Variational approach to factor analysis and related models, M.S. thesis 2004 Informatics and Mathematical Modelling, Technical University of Denmark, Lyngby, Denmark http://www2.imm.dtu.dk/pubdb/views/publication_details.php?id=3182

[B26] Ghahramani Z Beal MJ Solla SA, Leen TK, Müller K-R Variational inference for Bayesian mixtures of factor analysers Advances in Neural Information Processing Systems 1999 The MIT Press, Cambridge, Mass, USA 449 455

[B27] West M Bayesian factor regression models in the "large p, small n" paradigm Bayesian Statistics 2003 7 Oxford University Press, Oxford, UK 733 742

[B28] McLachlan GJ Bean RW Ben-Tovim Jones L Extension of the mixture of factor analyzers model to incorporate the multivariate -distribution Computational Statistics & Data Analysis 2007 51 11 5327 5338 10.1016/j.csda.2006.09.015

[B29] Huber W von Heydebreck A Sültmann H Poustka A Vingron M Variance stabilization applied to microarray data calibration and to the quantification of differential expression Bioinformatics 2002 18 supplement 1 S96 S104 10.1093/bioinformatics/18.suppl_1.S96 12169536

[B30] Fokoué E Titterington DM Mixtures of factor analysers. Bayesian estimation and inference by stochastic simulation Machine Learning 2003 50 1-2 73 94 10.1023/A:1020297828025

[B31] Green PJ Reversible jump Markov chain Monte Carlo computation and Bayesian model determination Biometrika 1995 82 4 711 732 10.1093/biomet/82.4.711

[B32] Ueda N Nakano R Ghahramani Z Hinton GE SMEM algorithm for mixture models Neural Computation 2000 12 9 2109 2128 10.1162/089976600300015088 10976141

[B33] Guelzim N Bottani S Bourgine P Képès F Topological and causal structure of the yeast transcriptional regulatory network Nature Genetics 2002 31 1 60 63 10.1038/ng873 11967534

[B34] Lee TI Rinaldi NJ Robert F Transcriptional regulatory networks in *Saccharomyces cerevisiae* Science 2002 298 5594 799 804 10.1126/science.1075090 12399584

[B35] Spellman PT Sherlock G Zhang MQ Comprehensive identification of cell cycle-regulated genes of the yeast *Saccharomyces cerevisiae* by microarray hybridization Molecular Biology of the Cell 1998 9 12 3273 3297 9843569 10.1091/mbc.9.12.3273 PMC25624

[B36] Gasch AP Spellman PT Kao CM Genomic expression programs in the response of yeast cells to environmental changes Molecular Biology of the Cell 2000 11 12 4241 4257 11102521 10.1091/mbc.11.12.4241 PMC15070

[B37] Mnaimneh S Davierwala AP Haynes J Exploration of essential gene functions via titratable promoter alleles Cell 2004 118 1 31 44 10.1016/j.cell.2004.06.013 15242642

[B38] Teixeira MC Monteiro P Jain P The YEASTRACT database: a tool for the analysis of transcription regulatory associations in *Saccharomyces cerevisiae* Nucleic Acids Research 2006 34, database issue D446 D451 10.1093/nar/gkj013 16381908 PMC1347376

[B39] Kaiser HF The varimax criterion for analytic rotation in factor analysis Psychometrika 1958 23 3 187 200 10.1007/BF02289233

[B40] Chang C Ding Z Hung YS Fung PCW Fast network component analysis (FastNCA) for gene regulatory network reconstruction from microarray data Bioinformatics 2008 24 11 1349 1358 10.1093/bioinformatics/btn131 18400771

[B41] Cowles MK Carlin BP Markov chain Monte Carlo convergence diagnostics: a comparative review Journal of the American Statistical Association 1996 91 434 883 904 10.2307/2291683

[B42] Bishop CM Pattern Recognition and Machine Learning 2006 Springer, Singapore

[B43] Friedman JH Meulman JJ Clustering objects on subsets of attributes (with discussion) Journal of the Royal Statistical Society: Series B 2004 66 4 815 849 10.1111/j.1467-9868.2004.02059.x

[B44] Lazzeroni L Owen A Plaid models for gene expression data Statistica Sinica 2002 12 1 61 86

[B45] Eisen MB Spellman PT Brown PO Botstein D Cluster analysis and display of genome-wide expression patterns Proceedings of the National Academy of Sciences of the United States of America 1998 95 25 14863 14868 10.1073/pnas.95.25.14863 9843981 PMC24541

[B46] Hastie T Tibshirani R Friedman J The Elements of Statistical Learning 2001 Springer, Berlin, Germany

[B47] Grossmann S Bauer S Robinson PN Vingron M Apostolico A, Guerra C, Istrail S, Pevzner PA, Waterman MS An improved statistic for detecting over-represented gene ontology annotations in gene sets Proceedings of the 10th Annual International Conference on Research in Computational Molecular Biology (RECOMB '06), Lecture Notes in Computer Science, Venice, Italy, April 2006 3909 Springer 85 98

[B48] Papoulis A Probability, Random Variables, and Stochastic Processes 1991 3 McGraw-Hill, Singapore

[B49] Grzegorczyk M Husmeier D Improving the structure MCMC sampler for Bayesian networks by introducing a new edge reversal move Machine Learning 2008 71 2-3 265 305 10.1007/s10994-008-5057-7

[B50] Jasra A Holmes CC Stephens DA Markov chain Monte Carlo methods and the label switching problem in Bayesian mixture modeling Statistical Science 2005 20 1 50 67 10.1214/088342305000000016

[B51] Kim TS Kim HY Yoon JH Kang HS Recruitment of the Swi/Snf complex by Ste12-Tec1 promotes Flo8-Mss11-mediated activation of *STA1* expression Molecular and Cellular Biology 2004 24 21 9542 9556 10.1128/MCB.24.21.9542-9556.2004 15485921 PMC522284

[B52] Barabási A-L Oltvai ZN Network biology: understanding the cell's functional organization Nature Reviews Genetics 2004 5 2 101 113 10.1038/nrg1272 14735121

[B53] Teh YW Jordan MI Beal MJ Blei DM Hierarchical dirichlet processes 2004 653 Department of Statistics, University of California, Berkeley, Calif, USA

[B54] Xing EP Jordan MI Sharan R Bayesian haplotype inference via the dirichlet process Journal of Computational Biology 2007 14 3 267 284 10.1089/cmb.2006.0102 17563311

[B55] Kuhn HW The Hungarian method for the assignment problem Naval Research Logistics 1955 2 1-2 83 97 10.1002/nav.3800020109

